# Sex-Specific Lifestyle and Biomedical Risk Factors for Chronic Disease among Early-Middle, Middle and Older Aged Australian Adults

**DOI:** 10.3390/ijerph16020224

**Published:** 2019-01-15

**Authors:** Sarah R. Dash, Erin Hoare, Pia Varsamis, Garry L. R. Jennings, Bronwyn A. Kingwell

**Affiliations:** 1 Metabolic and Vascular Physiology, Baker Heart and Diabetes Institute, Melbourne, VIC 3004, Australia; erin.hoare1@deakin.edu.au (E.H.); Pia.Varsamis@baker.edu.au (P.V.); garry.jennings@sydney.edu.au (G.L.R.J.); Bronwyn.Kingwell@baker.edu.au (B.A.K.); 2 Food and Mood Centre, Centre for Innovation in Mental and Physical Health and Clinical Treatment, School of Medicine, Faculty of Health, Deakin University, Melbourne, VIC 3004, Australia; 3 Sydney Medical School, University of Sydney, Sydney, NSW 2006, Australia

**Keywords:** risk factors, lifestyle, biomedical, chronic disease, sex

## Abstract

Evidence suggests age and sex differences in risk factors for chronic disease. This study examined lifestyle and biomedical risk factors among men (m) and women (w) in early-middle (25–51 years), middle (52–64) and older (65+) adulthood. Cross-sectional data from the 2011–2012 Australian Health Survey (n = 3024) were analysed. Self-reported dietary, activity, sleep behaviours and collected biomedical data were analysed. Early-middle adults failed to meet fruit, vegetable (95.3%) and sugar-sweetened beverage (SSB, 34.9%) recommendations. Older adults had higher prevalence of overweight/obesity (70%), high blood pressure (38.0%) and fewer met physical activity guidelines (36.3%). Prior to older adulthood, more men consumed SSBs (early-middle m 45.6%, w 24.4%; middle m 26.0%, w 19.3%), and fewer met sedentary behaviour recommendations (early-middle m 43.2%, w 62.1%; middle m 46.4%, w 63.9%). Differences in overweight/obese women in early-middle (44.8%) to middle adulthood (64.7%) were significant. Biomedical risk was greatest in middle age; abnormal cholesterol/lipids increased specifically for women (total cholesterol early-middle 24.9% middle 56.4%; abnormal LDL-cholesterol early-middle 23.1% middle 53.9%). Adherence to lifestyle guidelines was low; particularly among men. While men exhibited greater clinical risk overall, this significantly increased among women in middle-adulthood. Public health strategies to improve lifestyle, monitor and intervene among middle-aged women are warranted.

## 1. Introduction

Non-communicable diseases (NCDs) now account for 70% of deaths worldwide, and contribute an overwhelming majority of global disease burden [1,2]. The increase in NCDs has been linked with societal, environmental and lifestyle changes, and previous studies have highlighted the importance of modifiable factors in risk of disease [3,4]. NCD risk and onset is frequently underpinned by lifestyle behaviours, such as diet, physical activity, sedentary behaviour, smoking and alcohol consumption. Behavioural, environmental, occupational, and metabolic risks now explain half of global mortality, led by dietary and blood pressure risks [5].

Current evidence suggests that there are sex- and age-related differences in behavioural and biological risk factors for chronic disease [6]. Previous work has highlighted the importance of early life in establishing health behaviours and targeting early prevention and intervention efforts [7]. However, many NCD clinical risk factors emerge during adulthood, and primary prevention or management of chronic or comorbid conditions may be required [8]. The cross-sectional associations between health behaviours and risk of chronic disease in a sample of Australian adults has been previously observed [9]. Internationally, various surveys have reported risk factors for NCDs [10,11,12], or identified sex differences in health behaviours or mortality- and disability-adjusted life years [3,13]. While these studies have been important to tracking population health and understanding modifiable NCD risk, many have characterised risk by obesity status [14], or in relation to specific disease outcomes [15,16] which may preclude health tracking of the general population. As such, a general population, life course approaches to tracking national NCD risk may highlight patterns across a broad age-range of adults in the general population.

Building upon this, the current study includes both lifestyle and objective biomedical measures as indicators of risk for a range of chronic diseases, collected as part of the Australian Health Survey (AHS) and examines patterns in risk factor prevalence separately among men and women. Overall, this study aims to examine key lifestyle, clinical and biomedical characteristics among Australian adults in early-middle age (25–51 years), middle age (52–64 years) and older age (65+ years), and to examine sex differences in risk and protective factors.

## 2. Materials and Methods 

### 2.1. Study Design and Participants

The 2011–2012 Australian Health Survey (2011–2012 AHS) was a comprehensive, Australia-wide survey conducted by the Australian Bureau of Statistics to assess the health, risk factors, health service and medication usage of urban- and rural-dwelling Australians [17]. Briefly, the AHS comprised three surveys: National Health Survey (NHS), National Nutrition and Physical Activity Survey (NNPAS) and National Health Measures Survey (NHMS). The details of survey design and data collection provided here are further described elsewhere [18]. Briefly, the 2011–2012 AHS used a stratified, multistage area sample of private dwellings covering approximately 97% of Australia. The National Nutrition and Physical Activity Survey (NNPAS) formed one component the 2011–2012 AHS, which was conducted with a random subsample of residents within selected dwellings and included information on physical activity, sedentary behaviour and dietary intake [17]. Trained Australian Bureau of Statistics (ABS) interviewers conducted personal interviews in sample dwellings with selected residents over 18 years. The NHMS was a voluntary survey component, and all participants aged 12 and over were invited to provide blood and urine samples.

The current study includes individuals who completed the NNPAS and NHMS portions of the AHS. Participants included were aged 25 and over and those who fasted prior to blood collection (n = 3024). Data from the NHS component of the AHS was not available for this study and thus some measures (e.g., medication use) were not included in the current analysis.

### 2.2. Demographics

Age in years was self-reported and categorised by early-middle (25–51 years), middle (52–64 years) and older adulthood (65+ years). The Socio-Economic Indexes for Areas (SEIFA) were derived based on the area in which a participant lived, to determine socioeconomic advantage/disadvantage, and categorised by quintile [19].

### 2.3. Lifestyle Risk Factors

Usual daily serves of fruits and vegetables, sugar-sweetened beverage (SSB) and alcohol consumption was collected via 24-h recall. Current smoking and sleep duration on the day prior to interview was self-reported. Adherence to Australian adult physical activity (PA) guidelines was based on self-reported completion of five or more sessions of PA, for a total duration of 150 min of moderate-vigorous physical activity in a week [20]. Sedentary behaviour was based on self-report of total time (hours/day) during the day spent sitting or lying down for work, transport and leisure activities in the last week. 

### 2.4. Biomedical Risk Factors

All participants were invited to participate in the NHMS, and blood and urine samples were collected from those who consented [18]. In this cohort, all participants provided a fasting blood sample. Height and weight were measured during the survey interview, and body mass index (BMI) was calculated as weight (kilograms) divided by height (metres) squared. A BMI between 25 kg∙m^−2^ and 30 kg∙m^−2^ was categorised as overweight and BMI greater than 30 kg∙m^−2^ was categorised as obese [18]. These were combined as overweight/obese. Two blood pressure measurements were completed using an oscillometric automated blood pressure monitor that displayed systolic and diastolic pressures. A third reading was taken if there was significant difference (greater than 10 mmHg) between readings. Risk of diabetes was assessed using fasting plasma glucose (FPG) and glycated haemoglobin (HbA1c) tests. Blood lipid assessment included total cholesterol, high-density lipoprotein (HDL)-cholesterol and triglycerides. Low-density lipoprotein (LDL)-cholesterol was calculated using the Friedewald equation (LDL-cholesterol (mmol/L) = Total cholesterol − HDL-cholesterol − Triglycerides/2.2). To measure risk of chronic kidney disease, urinary albumin/creatinine ratio was calculated. Estimated glomerular filtration rate (eGFR) was calculated based on serum creatinine results in blood samples using the Chronic Kidney Disease Epidemiology Collaboration Equations. Liver function was assessed through blood levels of alanine aminotransferase (ALT) and gamma glutamyl transferase (GGT). All samples were assayed at the Douglass Hanly Moir (DHM) laboratory. The prevalence of abnormal biomedical parameters was reported based on AHS biomedical definitions (Table 1). 

### 2.5. Statistical Analyses

All statistical analyses were completed in Stata Corp, Stata/SE 14.0 (StataCorp LP., College Station, TX, USA) and weighted to account for the multistage sampling technique and to reflect the wider Australian population, using the biomedical weighting as per AHS recommendations [21]. Frequencies and weighted proportions were calculated for each age and sex category. Significance was calculated using binomial and multinomial logistic regression models, whereby age (early-middle, middle or older) and sex (male or female) exposure variables where entered as predictors of CVD lifestyle and biomedical risk factors. Unadjusted regression models were run for each age category among males and females separately for each demographic, lifestyle and biomedical outcome (Table 1). Significance was assumed at *p* < 0.05.

### 2.6. Ethics Approval

Permission was obtained from the Australian Bureau of Statistics to access de-identified Basic Confidential Unit Record Files (CURFs), released on November 2014 to conduct analysis for research purposes. Informed consent was obtained from all NHMS participants. Ethics approval for this study was granted from Alfred Health (Project No. 367/16).

## 3. Results 

**Figure 1:**
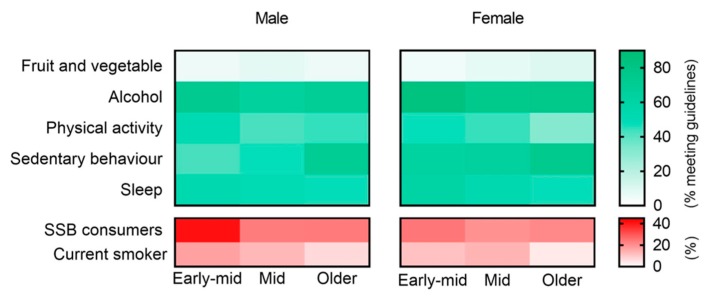
Proportion of Australian males and females meeting lifestyle guidelines, and proportion with lifestyle risk factors. Green panels show the percentage of individuals meeting guidelines (as defined in Table 1) and red panels show the percentage of individuals with specific adverse health behaviours. Abbreviations: SSB, sugar-sweetened beverage.

**Figure 2:**
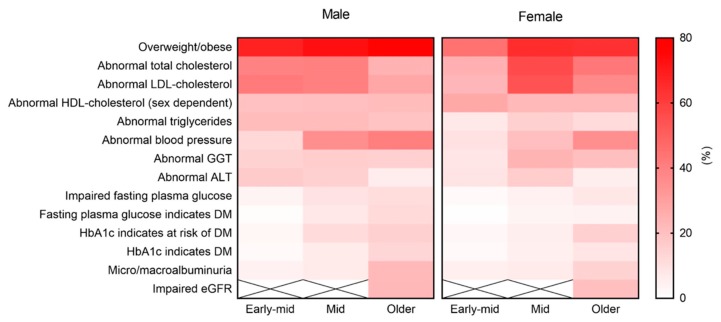
Proportion of Australian males and females with abnormal biomedical results throughout adulthood. Abbreviations: ALT, alanine aminotransferase; DM, diabetes mellitus; eGFR, estimated glomerular filtration rate; GGT, gamma-glutamyltransferase; HbA1c, glycated haemoglobin; HDL, high-density lipoprotein; LDL, low-density lipoprotein.

Demographic and lifestyle risk factors are reported in Table 2 and illustrated in Figure 1. Biomedical characteristics are reported in Table 3 and illustrated in Figure 2. 

### 3.1. Demographic and Lifestyle Risk Factors

The mean age of participants was 39 years for early-middle adults, 57 years for middle-adults and 72 years for older age adults. Level of disadvantage appeared evenly distributed, apart from men in the fourth SEIFA quintile (early-middle 23.1%, middle 17.0%).

Adherence to fruit and vegetable recommendations remained low (<10%) throughout adulthood, although a significantly higher proportion of older adults met the recommendations (7.6%) compared with early-middle adults (4.7%), specifically older females (9.7% vs. 4.2%). Early-middle adults were the highest consumers of SSBs (34.9%); nearly double the proportion of men consumed SSBs (45.6%) compared with women (24.4%), and consumption rates declined with increasing age categories. While fewer women consumed SSBs, the proportion of men consuming SSBs decreased significantly from early-middle (45.6%) to middle-adulthood (26.0%). The proportion adhering to alcohol consumption guidelines (max two standard drinks/day) was lowest in middle-adulthood (70%) and among men in each age category. 

Similarly, the proportion who met PA guidelines decreased with age, although differed by sex. For men, the proportion meeting PA guidelines decreased between early-middle (52.9%) and middle adulthood (41.9%). For women, the proportion meeting PA guidelines remained consistent in the two younger age groupings (46.0% early-middle vs. 43.3% middle), but decreased in the older group (30.8%). Older aged adults had the highest proportion meeting sedentary behaviour recommendations of less than 6 h/day (71.7%). The proportion meeting guidelines for sedentary time increased or remained stable with each increasing age category for both men and women, although fewer men met guidelines in each age category. Over half of adults in each age category met the sleep guideline of 7–9 h/night (early 56.9%; mid 51.4%; older 51.9%), and results were comparable between men and women in each category. The proportion of smokers decreased with age, and remained comparable between men and women. 

### 3.2. Biomedical Risk Factors

Overweight/obesity rates were greater with age, with a greater proportion of men overweight/obese in each age category. For women, the proportion that was overweight/obese was significantly greater among middle adults (64.7%) than early-middle adults (44.8%). Risk of diabetes increased with age for both sexes, however a higher proportion of men had elevated FPG and HbA1c indicating the presence of diabetes in middle (FPG 7.3% men, 3.4% women; HbA1c 6.3% men, 5.0% women) and older adulthood (FPG 12.0% men, 4.0% women; HbA1c 13.1% men, 8.3% women). For other markers of cardiovascular disease, the proportion of adults with abnormal blood pressure increased with age. A higher proportion of men had abnormal blood pressure in each age category, with the largest difference in middle adulthood (35.7% men, 20.4% women). For lipid biomarkers, the highest proportion of individuals with abnormal triglycerides (18.2%), LDL-cholesterol (47.0%) and total cholesterol (48.2%) was in middle adulthood. Additionally, the proportion of women with abnormal lipid biomarkers increased significantly from early-middle to middle adulthood, including abnormal LDL cholesterol (23.1% to 53.9%) and abnormal total cholesterol (24.9% to 56.4%). Proportions with abnormal HDL-cholesterol were similar across age categories, though slightly higher in females. Risk of kidney disease also increased with age among both sexes, although data for early-middle and middle adulthood are not shown, as per ABS confidentiality guidelines [18]. Abnormal liver function was highest in middle adulthood, with increases for women specifically in middle adulthood (GGT early–middle 8.0%, middle 23.9%; ALT early-middle 9.0%, middle 16.3%).

## 4. Discussion 

In general, patterns of adherence to lifestyle recommendations known to be protective against chronic disease risk differed between sexes across the adult age range. While adherence to lifestyle guidelines remained low across adulthood, certain detrimental lifestyle behaviours (i.e., smoking and SSB consumption) were less prevalent with age, whereas physical activity declined. Overall, men were inclined to exhibit a higher prevalence of risk behaviours than women (i.e., smoking, SSB and alcohol consumption and clinical risk factors such as overweight/obesity and abnormal blood pressure). The pattern of biomedical risk factors suggests the progression of disease with age from overweight/obesity, disrupted metabolism, through to organ failure. Interestingly, there were significant differences in biomedical risk profiles, particularly surrounding overweight/obesity, lipids and glucose metabolism, among women in early-middle and middle adulthood. These observations likely reflect the transition from pre- to post-menopausal between age categories [22]. These findings are concordant with previous work in the US [13], Europe [23], India [12], Brazil [11] and elsewhere [13,14] that has identified sex and age differences in risk factors for NCDs. Uniquely, the current study presents a life course approach to non-communicable disease risk and prevention, including a range shared risk factors for various NCDs that gives insight into sex-specific patterns between risk factors and disease progression.

### 4.1. Age and Sex Differences in Lifestyle

Given the aging global population, health behaviours are important in reducing health care costs and maintaining quality of life [24]. Concerningly, adherence to recommended health behaviours was particularly low in early-middle adulthood; an important phase of disease prevention and weight management. 

The current study is in agreement with previous research suggesting sex differences in health behaviours [25], as differing patterns in lifestyle risk factors emerged among men and women. A greater proportion of men are often reported as being physically active compared to women [26], and this was observed in the current sample, except during middle age. However, a greater proportion of women met the guidelines for sedentary behaviour in all age groups, suggesting that female may be more active during the day, whereas men may be more likely to do bouts of exercise. Further, a higher proportion of women met daily guidelines for alcohol intake, and fewer were overweight/obese in each age category. Various lifestyle factors improved across adulthood, and older adults were inclined to have fewer risk factors such as SSB consumption and smoking. Few older adults were adequately physical active, which may highlight the importance of age and ability appropriate guidelines among older adults. However, a larger proportion of older adults met the guidelines for sedentary behaviour, suggesting that older adults may partake in more light–moderate PA, which may be a suitable modification to PA guidelines among this age group.

Various social factors may contribute to sex-differences in health behaviours. For example, resting, leisure and occupational energy expenditure is often lower among women [27]. Evidence suggests that men may be less likely to report symptoms or illness [28]. Additionally, females may disproportionately face social barriers to physical activity, such as societal pressures surrounding body image, self-consciousness, or lack of time associated with undertaking both employment and household/caretaking responsibilities [29,30]. Conversely, risk taking behaviours have been associated with societal perceptions of masculinity, which may in turn influence health care practices and behaviours [31]. The sociocultural contextual influence in which health behaviours occur must be considered in health policy and public health action.

### 4.2. Age and Sex Differences in Biomedical Risk

Certainly, aging is associated with natural physiological changes that alter health [32]. In this study, biomedical risk factors for various chronic diseases were greater with older age. However, in some instances, biomedical risk factors were highest in middle adulthood. While the cross-sectional nature of this study does not allow for longitudinal analyses, it is possible that increased risk in middle age may be associated with lifestyle behaviours in earlier adulthood, as well as age-related changes in physiological processes. Additionally, improvements in biomedical risk factors in older adulthood may be explained by medication use, and are also likely to be related to a survival effect of participants in this category. These findings indicate a potential disease trajectory among Australian adults, beginning with overweight/obesity in early adulthood, to altered lipid, glucose and liver metabolism, progressing to vascular disease including hypertension and resulting in greater likelihood of end organ damage in later adulthood. This progression is likely underpinned by lifestyle behaviours, implying that current lifestyle and/or pharmacological approaches to disease management may be insufficient. 

In accordance with previous research, patterns of biomedical risk factors differed between sexes. Specifically, men tended to exhibit consistently higher proportions of abnormal biomedical parameters across age categories, whereas proportion of ‘at risk’ results tended to increase significantly between early-middle and middle adulthood in women. Previous epidemiological research has demonstrated that morbidity and mortality risks differ between sexes, whereby women have higher rates of disability and poorer self-rated health, and men have higher rates of mortality at every age [33]. 

Differences in observed biomedical risk among women across stages of adulthood may be underpinned by differences in overweight/obesity relative to reproductive stage. The transition from early-middle to middle adulthood was also characterised by lifestyle changes, such as decreases in the proportion of women meeting alcohol and guidelines, as well as an increase in proportion of overweight/obese. Hormonal changes during menopause are associated with changes in metabolism and fat distribution, which may increase risk of disease [34]. The average age of women currently experiencing menopause in this sample was 53, suggesting that the transition from early-middle to middle adulthood is likely marked by significant hormonal, compositional and metabolic changes that may increase certain risk factors [35]. Menopause related changes in glucose and lipid metabolism may increase risk of cardiometabolic disease among women. The results of this study suggest that lifestyle behaviours and biomedical status, as well as related factors such as medication, should be closely monitored during the menopausal transition.

### 4.3. Implications for Public Health—Guidelines, Prevention and Intervention

The public health implications of these findings are multiple. It could be argued that prevention messaging could be tailored to the unique sex- and age-specific needs of the Australian population. The social and cultural factors that shape health behaviours vary with age and it is unsurprising that clinical and lifestyle indicators of chronic disease also vary. Health outcomes are likely linked with risk behaviours from decades before and younger age groups may benefit from educational programs explaining such relationships. Older age groups face specific barriers, particularly when accessibility and mobility decrease with age. The current Australian PA guidelines for adults aged over 65 years recommend being physically active for 30 min every day [20]. However, these guidelines and related public health action may need to further consider individual circumstances and abilities, and address barriers to PA in old age. 

It is likely that as women progress through menopause, their experiences of behavioural, clinical and metabolic health are altered, and this period of time may require specific health support including closer monitoring and management of biomedical risk factors. The global financial burden of non-communicable disease is estimated to reach $13 trillion by 2030 [36], and there are potential economic benefits of further understanding the sex- and age-specific risks for chronic disease. When it is considered that physical inactivity, poor diet, and overweight/obese pose substantial global healthcare and economic burden, it is critical to understand determinants that may influence these lifestyle-related factors [37,38].

### 4.4. Strengths and Limitations

The current study is strengthened by the inclusion of multiple lifestyle factors associated with the risk of chronic disease, including sleep, diet, physical activity and sedentary behaviour. Though each of these is individually important to health, there is also likely to be a synergistic effect [39]. While the NNPAS took steps to minimise report bias, females may be more likely to misreport health and/or dietary habits based on social desirability [40]. However, the inclusion of objective biomedical measures in this study is not subject to the biases of self-report, and strengthens the examination of risk of chronic disease. Due to the components of the AHS available for this study, information on medication use was not available for this cohort. It is acknowledged that medication use is likely to impact biomedical results, particularly in the older age groupings, and this should be considered in the interpretation of results. Additionally, there are various factors that may contribute to NCD risk, including clustering of lifestyle risk factors that, while beyond the scope of the current study, should be considered. The 2011–2012 AHS was cross-sectional in design and thus conclusions on causality cannot be inferred, nor can individual risk be tracked across the life course. Despite this, the survey was the largest and most comprehensive ever conducted in Australia, and is population representative. Additionally, the examination of sex differences in lifestyle and biomedical risk factors highlights differing behavioural and biomedical trajectories between men and women across adulthood, and may highlight the potential value of targeted, sex-specific public health messages surrounding chronic disease prevention.

## 5. Conclusions

This study highlights relatively low overall adherence to protective, lifestyle recommendations among Australian adults. Importantly, there are various sex-differences in lifestyle behaviours associated with risk or chronic disease, as well as sex-specific patterns of biomedical risk factors across the adult age span. While men exhibited consistently higher prevalence of lifestyle and biomedical risk for chronic disease, females have differing risk prevalence according to life, and potentially, reproductive stage. As adulthood is often when chronic disease emerges, the surveillance of lifestyle and biomedical risk factors is of critical importance during this period. Taking a biopsychosocial approach, the role of gender, social expectations and norms cannot be separated from experiences of health, and should be considered in the preparation of effective public health messages and intervention.

## Figures and Tables

**Table 1:** Definitions of lifestyle and biomedical categorical variables.

Variable	Guideline/Definition
SEIFA	Index of relative socioeconomic disadvantage: 1 = Lowest 20%, 5 = Highest 20%
*Lifestyle Risk Factors*	
Fruit and Vegetable requirements	2 fruits and 5 vegetables per day
SSB consumers	Consumed SSB on day previous to interview, based on 24 h recall
Alcohol consumption	Under 20g alcohol/day (under 2 standard drinks for both men and women)
Physical Activity	150 min of physical activity over 5 or more sessions per week
Sedentary Behaviour	< 6 h/day of self-reported lying or sitting for work, transport or leisure activities
Sleep	7–9 h sleep/day
Current Smoker	Currently smoking cigarettes
*Biomedical Risk Factors*	
Impaired Fasting plasma glucose	>6.1 and <7.0 mmol/L
Fasting plasma glucose indicates diabetes	≥7.0 mmol/L
HbA1c, at risk of diabetes	>6.0 and <6.5%
HbA1c, has diabetes	≥6.5%
Abnormal blood pressure	140/90 to >180/110 mmHg
Abnormal triglycerides	≥2.0 mmol/L
Abnormal LDL cholesterol	≥3.5 mmol/L
Abnormal HDL cholesterol	Females < 1.3 mmol/L; Males < 1.0 mmol/L
Abnormal total cholesterol	≥5.5 mmol/L
Micro/macroalbuminuria (based on Albumin Creatinine Ratio (ACR)	Females microalbuminuria ACR ≥ 3.5 to ≤ 35.0 mg/mmol, Macroalbuminuria ACR > 35.0 mg/mmol; Males microalbuminuria ACR ≥ 2.5 to ≤ 25.0 mg/mmol, Macroalbuminuria ACR > 25.0 mg/mmol
Impaired estimated glomerular filtration rate (eGFR)	Impaired eGFR < 60 mL/min/1.73 m^2^
Abnormal gamma glutamyl transferase (GGT)	Females > 35 U/L; Males > 50 U/L
Abnormal alanine aminotransferase (ALT)	Females > 30 U/L; Males > 40 U/L

**Table 2:** Demographic and lifestyle risk factors of early-middle aged adults (21–51 years, n = 1397), middle aged-adults (52–63 years, n = 803) and older adults (65+ years, n = 824) in Australia *.

	Early-Middle Adult 21–51 Years			Middle-Adult 52–64 Years			Older Adult 65+ Years		
	Male n = 610 (49.5%) ^e^	Female n = 787 (50.4%)	Total n = 1397 (56.7%)	Male n = 364 (49.4%)	Female n = 439 (50.6%)	Total n = 803 (23.4%)	Male n = 369 (46.5%)	Female n = 455 (53.5%)	Total n = 824 (19.3%)
SEIFA index									
Lowest 20%	105 (16.6)	139 (17.2)	244 (16.9)	70 (17.8)	75 (18.8)	145 (18.3)	79 (22.8)	101 (21.2)	180 (21.9)
Second quintile	113 (17.9)	149 (17.9)	262 (17.9)	82 (20.2)	103 (21.0)	185 (20.6)	69 (18.2)	102 (18.6)	171 (18.4)
Third quintile	128 (22.0)	152 (19.3)	280 (20.6)	68 (18.4)	94 (22.9)	162 (20.7)	75 (19.2)	86 (20.0)	161 (19.6)
Fourth quintile	135 (23.1)	147 (22.0)	282 (22.5)	57 (17.0) ^b^	69 (16.5)	126 (16.7)	61 (17.6)	76 (17.4)	137 (17.5)
Highest 20%	129 (20.4)	200 (23.6)	329 (22.0)	87 (26.7)	98 (20.8)	185 (23.7)	85 (22.2)	90 (22.8)	175 (22.5)
***Lifestyle Risk Factors***									
Fruit and vegetable, n (% who met)	29 (5.3)	43 (4.2)	72 (4.7)	20 (7.4)	34 (6.6)	54 (7.0)	21 (5.1)	48 (9.7) ^b^	69 (7.6) **^a^**
SSB consumers, n (%)	257 (45.6)	188 (24.4)	445 (34.9)	98 (26.0) ^b^	92 (19.3)	190 (22.6) ^a^	94 (26.2) ^b^	94 (21.1)	188 (23.5) ^a^
Alcohol consumption, n (% who met)	420 (72.0)	638 (83.7)	1058 (77.9)	232 (64.3)	332 (75.6)	564(70.0)	248 (68.8)	368 (77.3)	616 (73.3)
Physical activity, n (% who met)	301 (52.9) ^d^	367 (46.0)	668 (48.7)	158 (41.9) ^b^	185 (43.3)	343 (42.6) ^a^	150 (42.5) ^b^	160 (30.8) ^b,d^	310 (36.3) ^a^
Sedentary behaviour, n (% who met)	261 (43.2)	476 (62.1)	737 (52.8)	172 (46.4)	273 (63.9)	445 (55.3)	244 (69.7) ^b,d^	328 (73.5) ^b,d^	572 (71.7) ^ac^
Sleep, n (% who met)	333 (53.9)	463 (59.8)	796 (56.9)	193 (49.9)	224 (52.8)	437 (51.4)	192 (47.4)	253 (55.9)	445 (51.9)
Current smoker, n (%)	114 (18.8)	112 (11.1)	226 (14.9)	55 (14.2)	56 (13.4)	111 (13.8)	26 (7.4) ^b,d^	30 (4.0) ^b,d^	56 (5.6) ^ac^

Significance was calculated using binomial and multinomial logistic regression models, whereby age (early-middle, middle or older) and sex (male or female) exposure variables where entered as predictors of CVD lifestyle and biomedical risk factors. Unadjusted regression models were run for each age category among males and females separately for each demographic, lifestyle and biomedical outcome. Significance was assumed at *p* < 0.05.^a^ Significantly different to early-middle adults (total). ^b^ Significantly different to early-middle adults (within sex). ^c^ Significantly different to mid adulthood (total). ^d^ Significantly different to mid adulthood (within sex). ^e^ Percentages relate to proportion of total sample within the column. * Weighted to reflect the wider Australian population. Abbreviations: SEIFA, Socio-Economic Indexes for Areas; SSB, sugar-sweetened beverage.

**Table 3:** Biomedical risk factors of early-middle adults (21–51 years, n = 1397), middle-adults (52–63 years, n = 803) and older adults (65+ years, n = 824) in Australia.

	Early-Middle Adult 21–51 Years			Middle-Adult 53–64 Years			Older Adult 65+ Years		
	Male n = 610 (49.5%) ^e^	Female n = 787 (50.4%)	Total n = 1397 (56.7%)	Male n = 364 (49.4%)	Female n = 439 (50.6%)	Total n = 803 (23.4%)	Male n = 369 (46.5%)	Female n = 455 (53.5%)	Total n = 824 (19.3%)
Overweight/Obese, n (%)	428 (67.8)	390 (44.8) ^d^	818 (56.2) ^c^	277 (72.5)	283 (64.7) ^b^	560 (68.6) ^a^	286 (77.3) ^b^	297 (63.7) ^b^	583 (70.0) ^a^
*Diabetes*									
Impaired Fasting Plasma Glucose (mmol/L), n (%)	17 (3.4)	15 (1.4)	32 (2.4)	24 (9.1) ^b^	22 (4.2) ^b^	46 (6.7) ^a^	49 (10.9) ^b^	37 (7.7) ^b^	86 (9.2) ^a^
Fasting Plasma Glucose (mmol/L) indicates diabetes, n (%)	9 (0.7)	9 (1.1)	18 (0.9)	31 (7.3) ^b^	15 (3.4)	46 (5.3) ^a^	43 (12.0) ^b^	26 (4.0) ^b^	69 (7.7) ^a^
HbA1c indicates at risk of diabetes, n (%)	19 (2.6)	14 (2.4)	33 (2.5)	34 (11.4) ^b^	29 (5.9) ^b^	63 (8.6) ^a^	58 (15.2) ^b^	73 (15.3) ^b,d^	131 (15.3) ^a,c^
HbA1c indicates diabetes (%), n (%)	12 (1.7)	13 (1.8)	25 (1.8)	22 (6.3) ^b^	21 (5.0) ^b^	43 (5.6) ^a^	48 (13.1) ^bd^	43 (8.3) ^b^	91 (10.5) ^ac^
*Cardiovascular Disease*									
Abnormal blood pressure, n (%)	97 (12.5) ^d^	78 (9.5) ^d^	175 (11.0)	132 (35.7) ^b^	91 (20.4) ^b^	223 (28.0) ^a^	154 (40.6) ^b^	149 (35.7) ^b,d^	303 (38.0) ^a,c^
Abnormal triglycerides (mmol/L), n (%)	128 (21.6)	67 (7.5) ^d^	195 (14.5)	96 (21.6)	71 (14.9) ^b^	167 (18.2)	69 (18.9)	61 (11.5)	130 (14.9)
Abnormal LDL-cholesterol (mmol/L), n (%)	256 (41.9)	188 (23.1) ^d^	444 (32.4)	154 (39.9)	228 (53.9) ^b^	382 (47.0) ^a^	103 (28.1) ^b,d^	182 (37.3) ^b,d^	285 (33.0) ^d^
Abnormal HDL-cholesterol (mmol/L), n (%) * sex dependent	109 (19.6)	222 (27.9)	331 (23.8)	75 (20.6)	98 (22.4)	173 (21.6)	75 (21.5)	105 (22.5)	180 (22.0)
Abnormal total cholesterol (mmol/L), n (%)	235 (39.5)	213 (24.9) ^d^	448 (32.1)	151 (39.9)	252 (56.4) ^b^	403 (48.2) ^a^	97 (24.3) ^b,d^	212 (42.8) ^b,d^	309 (34.2) ^d^
*Kidney Disease*									
Micro/macroalbuminuria (mg/mmol), n (%)	21 (4.1)	44 (4.9)	65 (4.5)	32 (6.9)	24 (6.9)	56 (6.9)	83 (22.2) ^b,d^	54 (14.2) ^b,d^	137 (17.9) ^a,c^
Impaired eGFR (mL/min), n (%) ^2^	-	- ^d^	-	-	-^b^	-^a^	73 (22.6) ^b,d^	71 (20.3) ^b,d^	144 (21.3) ^a,c^
*Liver Function*									
Abnormal Gamma glytamyl transferase (GGT) (U/L), n (%)	85 (13.9) ^d^	78 (8.0)	163 (11.0)	56 (15.7)	97 (23.9) ^b^	153 (19.8) ^a^	62 (14.8)	94 (20.2) ^b^	156 (17.7) ^a^
Abnormal Alanine aminotransferase (ALT) (U/L), n (%)	93 (16.9)	70 (9.0) ^d^	163 (12.9)	47 (14.9)	66 (16.3) ^b^	113 (15.6)	25 (6.1) ^bd^	25 (5.6) ^c^	50 (5.8) ^ac^

Significance was calculated using binomial and multinomial logistic regression models, whereby age (early-middle, middle or older) and sex (male or female) exposure variables where entered as predictors of CVD lifestyle and biomedical risk factors. Unadjusted regression models were run for each age category among males and females separately for each demographic, lifestyle and biomedical outcome. Significance was assumed at *p* < 0.05 ^a^ Significantly different to early-middle adults (total). ^b^ Significantly different to early-middle adults (within sex). ^c^ Significantly different to mid adulthood (total). ^d^ Significantly different to mid adulthood (within sex). ^e^ Percentages relate to proportion of total sample within the column ^1^ Percentages relate to proportion of total sample within the column. ^2^ Data not shown due to low cell value, as per AHS data disclosure recommendations [18]. *Weighted to reflect the wider Australian population. Abbreviations: ALT, alanine aminotransferase; DM, diabetes mellitus; eGFR, estimated Glomerular Filtration Rate; GGT, Gamma-glutamyltransferase; HbA1c, Glycated haemoglobin; HDL, high-density lipoprotein; LDL, low-density lipoprotein.

## Abbreviations

ALT	Alanine Aminotransferase
ABS	Australian Bureau of Statistics
AHS	Australian Health Survey
CURFS	Basic Confidential Unit Record Files
BMI	Body Mass Index
DHM	Douglass Hanly Moir
eGFR	Estimated Glomerular Filtration Rate
FPG	Fasting Plasma Glucose
GGT	Gamma-Glutamyl Transferase
HbA1c	Hemoglobin A1c
HDL	High-density Lipoprotein
LDL	Low-density Lipoprotein
NHMS	National Health Measure Survey
NHS	National Health Survey
NNPAS	National Nutrition and Physical Activity Survey
NCD	Non-communicable disease
PA	Physical Activity
SEIFA	Socio-Economic Indexes For Areas
SSB	Sugar-sweetened beverage
